# Supplementary material for: How trauma related to sex trafficking challenges parenting: Insights from Mexican and Central American survivors in the US
Source: PLoS One. 2021 Jun 16;16(6):e0252606. doi: 10.1371/journal.pone.0252606 (PMC8208566; doi:10.1371/journal.pone.0252606)
Supplement: S1 Table — (PDF) [file pone.0252606.s001.pdf]

Interview guide

History before and intra trafficking

Before we start talking about you and your child I would like to ask you some question about your life before being recluted in the trafficking net so I can understand where you come from and how was your life before.

1. Where did you grow up and with whom?
2. What is the best memory you have from your childhood
3. How was your relationship with your caretakers?
4. When did you leave your parents/caretakers house?
5. How old were you when you were recruited in the trafficking net? How were you recruited?
6. How old were you when you entered the US.
7. For how long you were trafficked before leaving the net?
8. How long it happen between you left and you became pregnant with your little child?

Now let's talk about you and your child.

Pregnancy

1. Sometimes, women plan to have a baby, other times they don't. Were you planning to have a baby when you became pregnant? Did your partner wanted to have this baby?
2. How did you feel when you found out that you were pregnant? (Probe: what made you feel like that)
3. How did you feel during your pregnancy? (Probe physical and mental helath)
4. What dreams did you have for this baby? (Probe: what were you excited about? Was there something that worried you?)
5. What was your experience giving birth? How did you feel when your baby was born? And the months after? (Probe: about different feelings, why or why not, how)
6. How was your child's father involved during the pregnancy? And after? How that made you feel?

The Child

1. Now, I'd like to begin by getting a sense of how you see your child... How would you describe him/her? (Ask for examples)
2. What would you describe as his/her favorite times?
3. And the times or things he has most trouble with?
4. What do you like most about your child? Why?
5. What do you like least about your child? Why?

The Relationship

1. How would you describe your relationship with your child? (Ask for memories that exemplify how she described the relationship)
2. What things you like to do together? (probe: do they play, sing songs, go for a walk, etc…)
3. Describe a time in the last week when you and (your child) felt really good together, very connected. (Probe: What made you feel like that?)

4. Describe a time in the last week when you and (your child) really were not feeling connected. (Probe: upset, or angry. What made you feel like that?)

Affective Experience of Parenting

Let's talk about how you feel as a mother

1. What gives you the most joy in being a mother? Why do you think that makes you feel joy?
2. What gives you the most pain or difficulty in being a parent? Why do you think that makes you feel like this? (alternative: What are the things about being a mom that are more challenging)
3. When you worry about (your child), what do you find yourself worrying most about? Why do you think you feel like this?
4. Did you ever feel angry or upset with your child? What kinds of situations make you feel this way? How do you handle your angry feelings?
5. Do you feel having your child changed you? How?
6. You have shared many experiences (reflect back to childhood experiences first and then trafficking) with me. You described these experiences as (use participants' words). How do you think your experiences affect how you feel as a mother now?
7. When you feel down or worried, what gives you strength to take care of yourself and take care of your baby/child?

Experiences with supports

1. Do you have any supports to take care of yourself and your child? (probe: partner, daycare, friend, family in US, family back home, sanctuary for families). How do you feel about these supports (probe: help with parenting, with health, with daily stressors)
2. In your everyday life, what are the things that you enjoy more doing? What makes you feel good? Why?
3. What are the things that are more challenging or that make you feel more stress? (probe: parenting, finances, mental health) Why?
4. What other supports you would like to have as a mother? (probe: financial supports, supports for the child, etc…). How these would help you feel better?

Thank you so much for taking the time to talk with us today.
